# A Purified Biflavonoid Extract From *Selaginella moellendorffii* Alleviates Gout Arthritis via NLRP3/ASC/Caspase-1 Axis Suppression

**DOI:** 10.3389/fphar.2021.676297

**Published:** 2021-05-17

**Authors:** Xueyan Zhang, Yingbo Liu, Guangrui Deng, Bisheng Huang, Guoyin Kai, Keli chen, Juan Li

**Affiliations:** ^1^ Key Laboratory of Ministry of Education on Traditional Chinese Medicine Resource and Compound Prescription, Key Laboratory of Resources and Chemistry of Chinese Medicine, College of Pharmacy, Hubei University of Chinese Medicine, Wuhan, China; ^2^ Department of Pharmacy, Huanggang Hospital of Traditional Chinese Medicine, Huanggang, China; ^3^ College of Pharmacy, Zhejiang Chinese Medical University, Hangzhou, China

**Keywords:** *Selaginella moellendorffii*, biflavonoid, amentoflavone, NLRP3, gout

## Abstract

**Background:** Activation of nucleotide oligomerization domain-like receptor protein 3 (NLRP3) inflammasome plays a crucial role in gout. *Selaginella moellendorffii* has been confirmed effective for the treatment of gout in hospital preparations. Flavonoids, such as amentoflavone (AM), are the main active components of this medicine.

**Purpose:** We aimed to investigate the flavonoid extract (TF) and AM's effects on NLRP3 inflammasome *in vitro* and their preventive effects on gout *in vivo*.

**Methods:** LC-MS method was employed to investigate the chemical profile of TF. The cellular inflammation model was established by lipopolysaccharide (LPS) or monosodium urate (MSU) stimulation. The cell membrane integrality and morphological characteristics were determined by using Lactate dehydrogenase (LDH) assay kits, propidium iodide (PI) stain, and scanning electron microscopy (SEM). The inflammatory cytokines and NLRP3 inflammasome activation were determined using enzyme-linked immunosorbent assay (ELISA), quantitative real-time polymerase chain reaction (RT-PCR), immunofluorescence staining, and western blotting. The acute gout mouse model was induced by MSU injection into footpads, and then the paw edema, inflammatory mediators, and histological examination (HE) were analyzed.

**Results:** The main constituents in TF are AM and robustaflavone. In the cellular inflammation model, TF down-regulated the levels of nitric oxide (NO), TNF-α, and LDH, suppressed NLRP3 inflammasome-derived interleukin-1β (IL-1β) secretion, decreased caspase-1 activation, repressed mature IL-1β expression, inhibited ASC speck formation and NLRP3 protein expression. In an acute gout mouse model, oral administration of TF to mice effectively alleviated paw edema, reduced inflammatory features, and decreased the levels of IL-1β in mouse foot tissue. Similarly, the characteristic constituent AM was also able to down-regulated the levels of NO, TNF-α, and LDH, down-regulate the mRNA expression of IL-1β, TNF-α, caspase-1, and NLRP3. Besides, the foot thickness, lymphocyte infiltration, and IL-1β level were also prevented by AM.

**Conclusion:** The results indicated that TF and its main constituent AM alleviate gout arthritis via NLRP3/ASC/Caspase-1 axis suppression.

## Introduction

Gout is the most common inflammatory arthritis characterized by chronic elevation of serum uric acid levels above a deposition threshold to form monosodium urate (MSU) crystal in joints or tissues (Kuo et al., 2015). The incidence of gout has been increasing in recent years due to changes in lifestyles. Generally, it occurs in men 2–6 folds higher than in women (Ragab et al., 2017). The acute attack of gout causes a severe inflammatory response, including redness, hotness, tenderness, and swelling (Ragab et al., 2017). If left untreated, it might ultimately progress to chronic arthritis, tophi depositions, as well as renal stones (Lu et al., 2019). The attack of gout seriously impairs the quality of life of patients.

The precipitation of MSU crystals in joints is considered a crucial factor in gout initiation and development (Shi et al., 2010). Some evidence has demonstrated that the MSU crystal-induced inflammatory responses and gout pathogenesis are dependent on NLRP3 inflammasome activation (Szekanecz et al., 2019; Punzi et al., 2020). The NLRP3 inflammasome, composed of receptor protein NLRP3, adaptor protein ASC, and effector protein pro-caspase-1, is a multiprotein complex of more than 700 kDa (Petrilli et al., 2005). NLRP3 inflammasome can be activated by many different stimuli, including pathogen-associated molecular patterns (PAMPs) and danger-associated molecular patterns (DAMPs), such as MSU (Kingsbury et al., 2011; Guo et al., 2015). Once sensing specific activators, the sensor molecule NLRP3 undergoes conformational changes and then catalyzes ASC oligomerization to form the ASC “speck” (Vajjhala et al., 2012). As a macromolecular signaling platform, speck-like protein ASC then recruits the pro-caspase-1 via its C-terminal CARD domain, allowing for pro-caspase-1 is cleaved to generate caspase-1(Srinivasula et al., 2002). Caspase-1 cleaves the pro-IL-1β and pro-IL-18 precursor into mature IL-1β and IL-18, leading to inflammation and tissue destruction (Fink and Cookson, 2006). The aberrant activation of the NLRP3 inflammasome is related to various diseases, including arthritis, cardiovascular disease, diabetes, and other rare genetic disorders (Dixit, 2013; Rowczenio et al., 2017; Yang et al., 2018; Zhou et al., 2018). Given the large number and diversity of NLRP3 inflammasome activators, searching for inhibitory agents targeting NLRP3 inflammasome might be a better choice for the treatment of NLRP3 inflammasome-related diseases.

*Selaginella moellendorffii* Hieron, a perennial herb of the *Selaginella* genus, is used as an ethnic drug to treat idiopathic thrombocytopenic purpura and hepatitis (Shi et al., 2008). *S*. *moellendorffii* are known to be rich in bioflavonoids (Cao et al., 2010), such as amentoflavone, robustaflavone, bilobetin, hinokiflavone, podocarpusflavone A, and ginkgetin, which are responsible for the anti-inflammatory and immunomodulatory effects (Lee et al., 1995; Lee et al., 1997). The potent ethnopharmacological properties of *S*. *moellendorffii* make it an excellent natural source of novel medicinal targets for NLRP3 inflammasome-related gout treatment. In a previous study, the hospital preparation developed by our research group with the raw material of *S*. *moellendorffii* has achieved an excellent clinical effect on gout treatment. Its mechanism may be relevant to suppress NF-κB P65 activation and inflammatory mediator IL-1β secretion (Zhang et al., 2019). These findings prompted us to investigate the possible mechanism of flavonoids in controlling NLRP3 inflammasome activation. In continuation with our previous work, the present study demonstrated the TF from *S*. *moellendorffii* exerted its anti-gout activity by inhibiting the NLRP3/ASC/Caspase-1 pathway.

## Materials and Methods

### Reagent

AM (P09J8F37712) were obtained from Yuanye Bio-Technology (Shanghai, China), its purity was ≥99.0%; LPS (L4391), MSU (U2875), and phorbol myristate acetate (PMA, P8139) were purchased from Sigma-Aldrich (St. Louis, United States). PI (E24567B117) and hoechst 33,342 (EZ5679A169) were purchased from BioFRoxx (Einhausen, DE). Colchicine (COL, C106740) was from Aladdin (Shanghai, China). TNF-α and IL-1β enzyme-linked immunosorbent assay (ELISA) kits were purchased from Jiancheng Bioengineering Institute (Nanjing, China). LDH and NO assay kits were purchased from Beyotime (Shanghai, China). Antibodies of β-actin, caspase-1, IL-1β, NLRP3, and ASC were obtained from ABclonal (Wuhan, China). FITC-conjugated goat anti-rabbit IgG (GB22303) and HRP-conjugated goat anti-rabbit IgG were bought from Servicebio (Wuhan, China). All cell culture reagents were bought from Hyclone.

### Plant Material

Intact plants of *S. mollendorffii* were purchased from Jointown Pharmaceutical Group Co., Ltd. (Wuhan, China), and specimens of these materials (S190712) were deposited in Hubei University of Chinese Medicine’s herbarium. The plant material was authenticated by authors (Prof. Keli Chen).

### TF Preparation and Chemical Elucidation

The dry plant material (5 kg) was crushed and extracted with 85% ethanol for 1 h three times. The extract was filtered and then concentrated under the reduced pressure condition. The concentrated solution defatted with petroleum ether and then extracted with ethyl acetate. The ethyl acetate fraction (60 g) was successively dissolved with warm water and then subjected to D101 macroporous resin column chromatography eluted with 25, 50, 75, 95% alcohol (V/V) in a gradient manner. The different fractions were collected and evaporated. Finally, according to the previous methods (Yin and Chen, 2010), the flavonoid contents of different fractions were detected by UV spectra. The 50% ethanol fraction has total flavonoid content of 57.77 ± 4.23% and showed the strongest anti-inflammation activity by decreasing NO secretion of RAW264.7 cells in response to LPS. This fraction was used as the purified flavonoid extract (TF, 6.3 g) in this research.

The AM standard and TF were analyzed on Agilent 1260 Infinity Ⅱ HPLC system and 6465B MS system. Separations were accomplished on an Agilent RRHD Eclipse Plus C18 (2.1 mm × 50 mm, 1.8 μm). The mobile phase consisted of acetonitrile (A) and water, including 0.1% formic acid (B). The degree program was 35–60% A in 1–15 min. The flow rate was 0.2 ml/min, and the column temperature was maintained at 30°C. The injection volume was 2 μL. The detector was set at 330 nm for acquiring chromatograms.

The mass spectrometer was operated in positive ion mode with an AJS ESI source. The parameters were as follows: capillary voltage at 4000 V, nozzle voltage at 1500 V, fragmentor voltage at 100 V. High-purity nitrogen was utilized as a sheath gas. The flow rate was set at 11 L/min and gas temperature at 350°C. The collision energy (CE) was set at 45 V. The MS^2^ scan was in the range of 50–500 m*/z*, and the sweeping time was 500 ms.

### Cell Culture and Treatments

#### Cell Culture and Stimulation

The mouse macrophage cell line RAW 264.7 and the human monocytic cell line THP-1 were provided by the China Center for Type Culture Collection of Wuhan University (Wuhan, China). RAW264.7 cells were cultured in Dulbecco’s modified Eagle medium (DMEM) supplemented with 10% fetal bovine serum (FBS) and 1% streptomycin/penicillin at 37°C with 5% CO_2_. For treatment, the cells were pretreated with TF and AM for 2 h and subsequently treated by LPS (0.5 μg/ml) for 24 h.

THP-1 cells were grown in RPMI 1640 medium supplemented with 10% FBS and 1% streptomycin/penicillin. Before experiments, THP-1 cells were differentiated in a medium containing 100 nM PMA for 12 h, and then rested in a fresh medium for 1 day. For inducing NLRP3 inflammasome activation, THP-1 macrophages were seeded in 24-well or 6-well plates overnight, then the medium was changed to Opti-MEM, cells were primed with LPS (0.5 μg/ml) for 3 h, TF and AM were added for a further 1 h, and finally, cells were stimulated with MSU (150 μg/ml) for 6 h.

#### Cell Viability Assay

The cell counting kit-8 (CCK-8) assay was used to evaluate viability quantitatively. Briefly, the cells were seeded into 96-well plates. After incubation with a series of concentrations of test drugs diluted with culture medium for 24 h, the medium was removed, 10% CCK-8 reagent was added and incubated with cells for an additional 2 h. Finally, a microplate reader (BioTek Instruments, Inc, United States) was used to measure the absorbance at a wavelength of 450 nm.

#### Inflammatory Cytokine Determination

The cell supernatants were collected and stored at -80°C, then assayed for NO, IL-1β, and TNF-a. According to the manufacturer’s instructions, NO production was estimated according to the Griess method, whereas the quantification of IL-1β and TNF-a was performed using ELISA kits.

#### LDH Release Assay

After the THP-1 cells were treated mentioned above, the culture supernatants were harvested, and LDH levels were assessed by LDH cytotoxicity assay kit according to the manufacturer’s instructions (Beyotime, Shanghai, China).

#### SEM

THP-1 cells were grown on round glass coverslips pre-coated with 1% poly-l-lysine. After treatment, the cell fixed with 2.5% glutaraldehyde overnight and then washed with PBS three times. The sample was dehydrated through ethanol series (30, 50, 70, 95, and 100%), then dried by tertiary butanol method. Dried specimens were coated with gold-palladium and imaged with SEM operating at 30 kV (JSM-6510LV; JEOL, Tokyo, Japan).

#### RNA Preparation and RT-PCR

Total RNA was extracted from THP-1 cells with Trizol kit (Tiangen, China), then the RNA was reverse transcribed into cDNA using the FastKing Kit (Tiangen, China). The relative levels of target gene mRNA to the control β-actin were determined by using SYBR Green PCR master mix and analyzed with a Light Cycler 480 (Roche, Germany). The primers were designed and composed by Sangon Biotech (shanghai) as follows (Table 1). RT-PCR was performed under the following conditions: one cycle of 95°C for 15 min, and subjected to 40 cycles of 95°C for 10 s, 60°C for 40 s and 72°C for 32 s. Duplicate assays were performed with all samples. The expression of genes was calculated using the ΔΔ Ct method and expressed as fold change. RT-PCR products were resolved on 2.5% agarose gel and visualized with UV light after being stained with Green-DNA Dye.

**TABLE 1:** The specific primers used in RT-PCR assay (Human).

Gene name	Primer sequence (5′-3′)	Length (bp)
IL-1β	Forward: GAA​TCT​CCG​ACC​ACC​ACT​A	190
	Reverse: ACA​TAA​GCC​TCG​TTA​TCC​C	
NLRP3	Forward: AAC​AGC​CAC​CTC​ACT​TCC​AG	169
	Reverse: CCA​ACC​ACA​ATC​TCC​GAA​TG	
Caspase-1	Forward: GCA​CAA​GAC​CTC​TGA​CAG​CA	146
	Reverse: TTG​GGC​AGT​TCT​TGG​TAT​TC	
TNF-α	Forward: CTG​GTA​TGA​GCC​CAT​CTA​TC	296
	Reverse: CGA​AGT​GGT​GGT​CTT​GTT​GC	
β-actin	Forward: CCT​GAC​TGA​CTA​CCT​CAT​GAA​G	106
	Reverse: GAC​GTA​GCA​CAG​CTT​CTC​CTT​A	

#### Hochest 33342 and PI Double Staining

THP-1 cells (2.5 × 10^6^/well) were cultured in a 6-well plate and treated with test drugs mentioned above. Cells were stained with Hoechst 33342 (10 μg/ml, staining for all cells) for 20 min and propidium iodide (PI; 10 μg/ml, staining for membrane-damaged cells) for 10 min in the dark at 37°C. After that, the stained cells were observed under a fluorescence microscope (IX73; Olympus, Tokyo, Japan).

#### Immunofluorescence

Immunofluorescence was used to measure ASC speckles and NLRP3 protein expression. The cells were fixed with 4% buffered paraformaldehyde for 20 min, then permeabilized with 0.2% Triton-X for 10 min. After being washing several times, the cells were blocked with 6% goat serum in PBS for 1 h, and incubated with the primary antibody (1:100 dilution) at 4°C overnight. Subsequently, the cells were incubated with FITC-conjugated anti-rabbit IgG (1:100 dilution) for 1 h in the dark at room temperature. DAPI was used for counterstaining the nuclei for 7 min, and fluorescence was visualized using a fluorescence microscope at a magnification of × 200. NLRP3 fluorescence intensity and ASC speckles were quantified using ImageJ software.

#### Western Blot Assay

THP-1 cells were lyzed on ice with RIPA lysis buffer. After quantifying protein concentration using a BCA kit, equal amounts of protein samples (20 µg per lane) were separated by 12% SDS-PAGE and transferred onto PVDF membranes (Roche, Germany). The membranes incubated with primary antibodies (1:1000) diluted in 5% (w/v) fat-free milk at 4°C overnight. After being washed and incubated with horseradish peroxidase-conjugated secondary antibodies, the protein bands were visualized using an enhanced chemiluminescence (ECL) Western blotting kit (Meilun, China) and densitometrically evaluated with ImageJ software. β-actin served as the internal standard.

### Animals and Treatments

#### Animals

Specific pathogen-free C57BL6 male mice weighing between 20 and 22 g, were purchased from Changsheng biotechnology co, Ltd. (Liaoning, China). The Laboratory animal permission number was SCXK (Liao) 2020–0001. All mice were housed under standard laboratory conditions, including the air temperature 22 ± 2°C and relative humidity 50 ± 10% with a 12 h light-dark cycle. The experimental protocol in this study was approved by the Animal Ethical Committee of Hubei University of Chinese medicine (permission number: SYXK 2017-0067).

#### Induction of Gouty Arthritis With MSU Crystals in Mice

**FIGURE 1:**
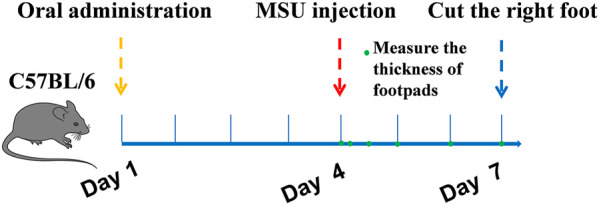
Schematic experimental protocols.

The gout model was performed as previously described (Dhanasekar and Rasool, 2016; Lu et al., 2019). C57BL6 mice were divided into six groups (*n* = 10): normal control group, MSU control group, colchicine group (2 mg/kg/d), TF group (400, 200, 100 mg/kg) and AM group (80, 40, 20 mg/kg). Besides the normal control group, injected with 50 μL sterile endotoxin-free saline, each group was subcutaneously injected with 50 μL suspension of MSU (50 mg/ml) under the plantar surface of the right paw. Mice were orally fed with corresponding samples for one week. The control group and MSU group were also treated with 0.9% normal saline. Inflammatory paw edema was quantified by measuring the thickness of the paw with a Vernier scale at 0, 4, 12, 24, and 72 h after MSU crystal injections (Figure 1). The minimum accuracy of which is 0.01 mm. Edema formation was described as the circumstance difference (Δmm) between the basal value and the test value. At the end of the experimental period, all mice were anesthetized, and the foot samples were removed and stored at -80°C. The foot tissues were homogenized in 0.1 M phosphate buffer (pH 7.2), and the supernatants were collected for IL-1β assays. For histological analysis, sagittal sections of the footpads were fixed in 4% paraformaldehyde, embedded in paraffin, and then stained with HE using standard techniques.

### Statistical Analysis

All measurement data were expressed as mean ± standard error of the mean (SEM). Differences among multiple groups were assessed by one-way analysis of variance (ANOVA) and Newman-Keuls Multiple Comparison Test using the GraphPad Prism 5. *p* < 0.05 was considered statistically significant.

## Results

### Chemical Characterization of TF

**FIGURE 2:**
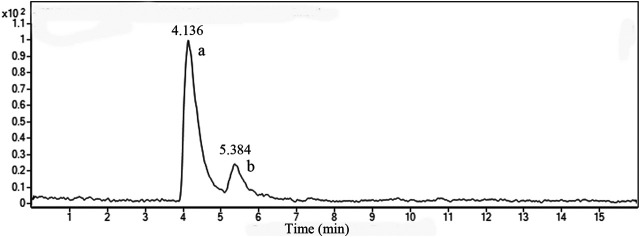
TIC of TF in positive mode. a, amentoflavone; b, robustaflavone.

Figure 2 shows the LC-MS total ion chromatograms (TIC) of TF in positive ESI-MS^E^ mode. Two prominent peaks appeared in the chromatograms and their retention times (RT) were 4.136 and 5.384 min, respectively. The quasi-molecular ion [M + H]+all at *m/z* 539.1002 in the positive ESI mode suggested the molecular formula C_30_H_18_O_10_. According to the standard compound peak, we initially speculated the peak a was AM. Based on the main fragmentations of mass spectrums in positive ESI-MS^E^ mode and related literature (Wang et al., 2015), we deduced that peak b was most likely robustaflavone.

### Effects of TF and AM on Cytotoxicity in RAW264.7 Cells and THP-1 Cells

**FIGURE 3:**
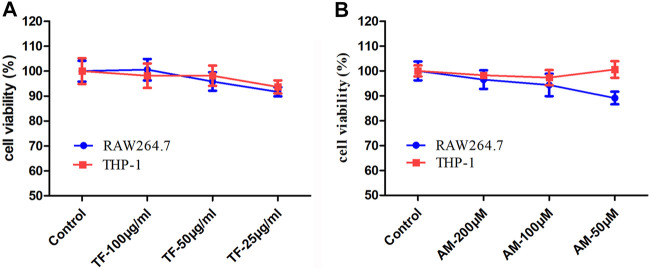
The effects of TF and AM on cytotoxicity in RAW264.7 cells and THP-1 cells. **(A)** Viability of RAW264.7 cells and THP-1 cells treated with TF. **(B)** Viability of RAW264.7 cells and THP-1 cells treated with AM. TF, flavonoid extract; AM, amentoflavone.

As shown in Figure 3, the TF at a dose up to 100 μg/ml caused no cytotoxicity in RAW264.7 cells and THP-1 cells during 24 h treatment. Additionally, AM did not affect the cell viability in RAW264.7 cells and THP-1 cells up to 200 μM (*p* > 0.05).

### Effects of TF and AM on Inflammatory Cytokine in LPS-Induced RAW 264.7 Cells and LPS/MSU-Induced THP-1 Macrophages

**FIGURE 4:**
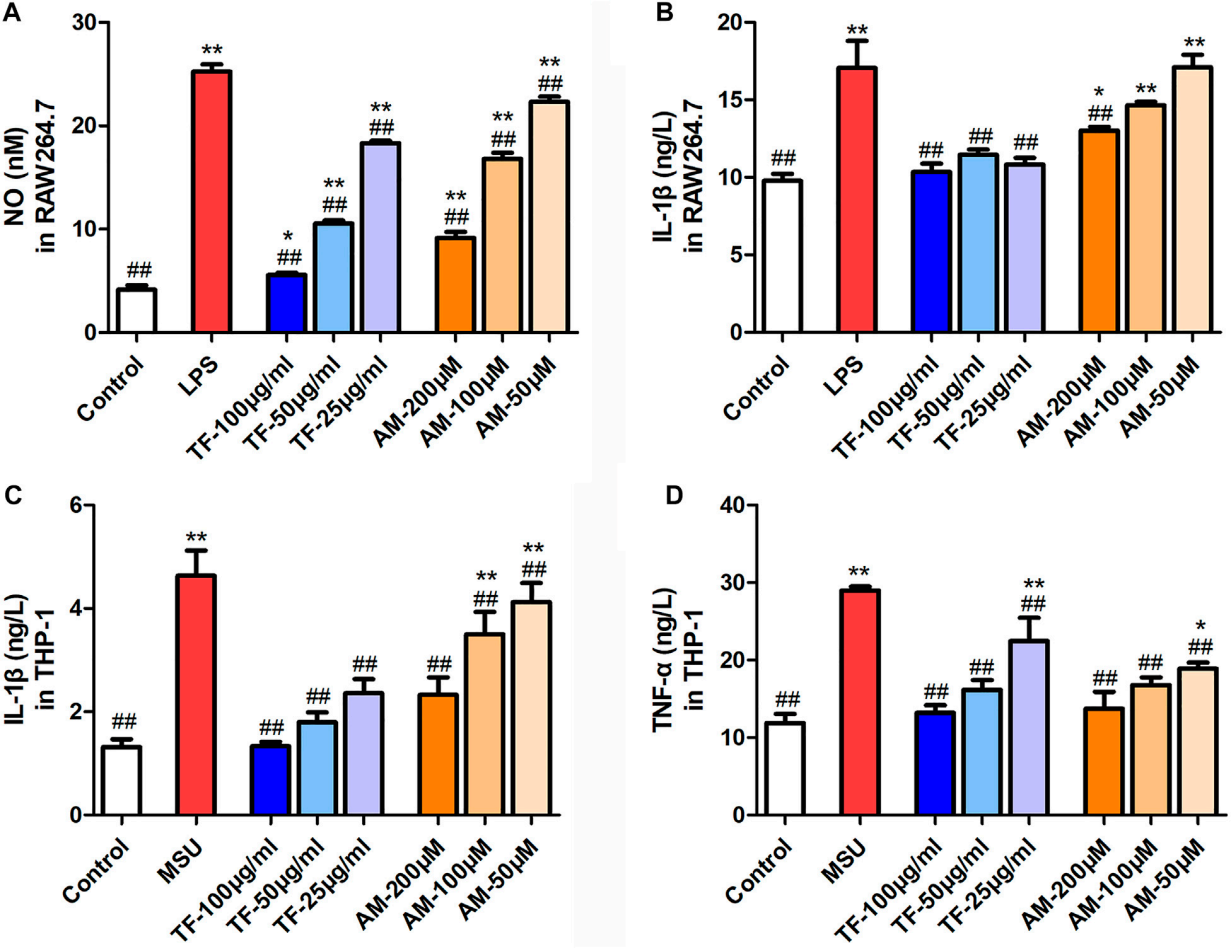
The effects of TF and AM on inflammatory cytokine **(A)** The level of NO in the RAW264.7 cells **(B)** The level of IL-1β in the RAW264.7 cells **(C)** The level of IL-1β in the THP-1 cells **(D)** The level of TNF-α in the THP-1 cells. *p < 0.05 and **p < 0.01, significantly different the normal control group; ^#^ *p* < 0.05 and ^##^ *p* < 0.01, significantly different the LPS/MSU control group. TF, flavonoid extract; AM, amentoflavone.

RAW 264.7 cells in the resting state released 4.13 ± 0.87 nM of NO during incubation for 24 h, whereas the cells markedly increased NO production up to 25.23 ± 1.44 nM in response to LPS stimulation. When cells were pretreated with TF and AM, NO production was dose-dependently inhibited (Figure 4A). As expected, TF and AM also decreased the level of IL-1β as compared with the LPS group (*p* < 0.05). In the meanwhile, IL-1β and TNF-α were determined in culture supernatant from the LPS/MSU-induced THP-1 cells. As shown in Figures 4C,D 25‐100 μg/mL TF and 50–200 μM AM could greatly down-regulate the levels of IL-1β and TNF-α (*p* < 0.01).

### Effects of TF and AM on Membrane Integrality in LPS/MSU-Induced THP-1 Macrophages

**FIGURE 5:**
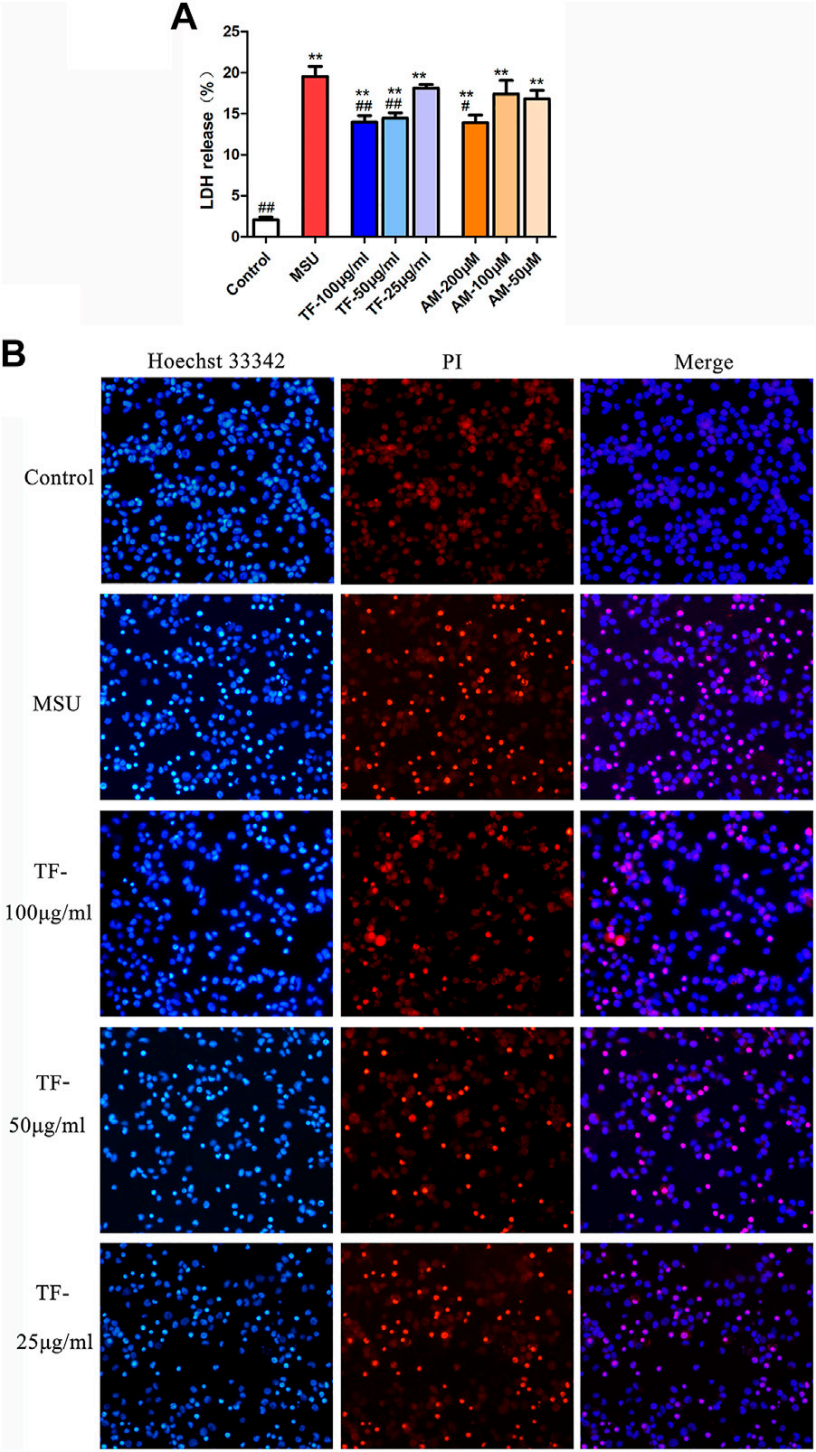
Effects of TF and AM on membrane integrality in LPS/MSU-induced THP-1 cells **(A)** The rate of LDH release level. **(B)**. Hochest33342 and PI double staining images were captured by fluorescence microscopy (200 ×). Red shows membrane-damaged cells. Blue shows nuclei. *p < 0.05 and **p < 0.01, significantly different the normal control group; ^#^ *p* < 0.05 and ^##^ *p* < 0.01, significantly different the LPS/MSU control group. TF, flavonoid extract; AM, amentoflavone.

To further assess the influence of membrane integrality on LPS/MSU-induced THP-1 cells pretreated with TF and AM, LDH release assay and Hochest 33342 and PI double staining were measured. As shown in Figure 5A, the LPS/MSU-induced LDH release significantly increased compared to the control group (*p* < 0.01), while the increase in LDH level was prevented by both TF and AM (*p* < 0.05, *p* < 0.01). Besides, the percentage of membrane-damaged THP-1 macrophages (red) to the total cells (blue) upregulated after LPS/MSU stimulation, yet TF could partly downregulate the ratio of PI-positive cells in a dose-dependent manner (Figure 5B).

### Effects of TF on Surface Morphology in LPS/MSU-Induced THP-1 Macrophages

**FIGURE 6:**
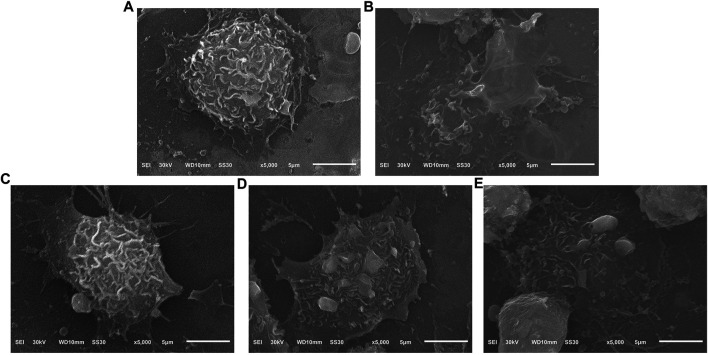
Effects of TF on surface morphology in LPS/MSU-induced THP-1 macrophages (× 5000). **(A)** Control group; **(B)** LPS/MSU group; **(C)** TF-100 μg/ml group; **(D)** TF-50 μg/ml group; **(E)** TF-25 μg/ml group. Scale bar, 5 µm. TF, flavonoid extract.

SEM analysis showed that THP-1 macrophages of the control group maintained a spherical morphology and a normal roughened surface, with only a few small extended lamellipodia (Figure 6A). By contrast, in LPS/MSU group, the cell membranes had lost their integrity. The cells showed mostly extended lamellipodia from the entire periphery of the cell and remained tightly attached to the culture slide, followed by cytoplasm flattening (Figure 6B). These characteristics were consistent with the late pyroptosis as previously reported (Chen et al., 2016b). After 100 μg/mL TF treatment, the cell contours became clearer, and surface villi became denser, indicating that pyroptosis characteristics were significantly reduced (Figures 6C–E).

### Effects of TF and AM on mRNA Expression in LPS/MSU-Induced THP-1 Macrophages

**FIGURE 7:**
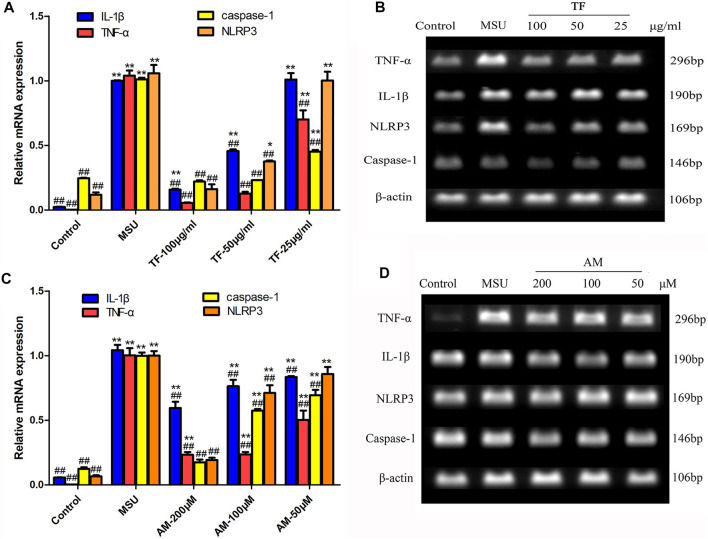
The Effects of TF and AM on mRNA in LPS/MSU-induced THP-1 macrophages. **(A, C)** The gene expressions of TNF-α, IL-1β, NLRP3 and caspase-1 mRNA **(B, D)** The detected bands of gene products. *p < 0.05 and **p < 0.01, significantly different the normal control group; ^#^ *p* < 0.05 and ^##^ *p* < 0.01, significantly different the LPS/MSU control group. TF, flavonoid extract; AM, amentoflavone.

To explore the underlying anti-inflammatory mechanisms, we investigated the inflammatory mediators including TNF-α, IL-1β, NLRP3, and caspase-1 in LPS/MSU-induced THP-1 macrophages. As shown in Figure 7, compared with the control group, the mRNA levels of TNF-α, IL-1β, NLRP3, and caspase-1 were significantly increased in the MSU group (*p* < 0.01). Conversely, pretreatment with TF and AM showed a significantly lower transcriptional level than those in the MSU group (*p* < 0.05).

### Effects of TF on ASC Speckle Formation and NLRP3 Expression in LPS/MSU-Induced THP-1 Macrophages

**FIGURE 8:**
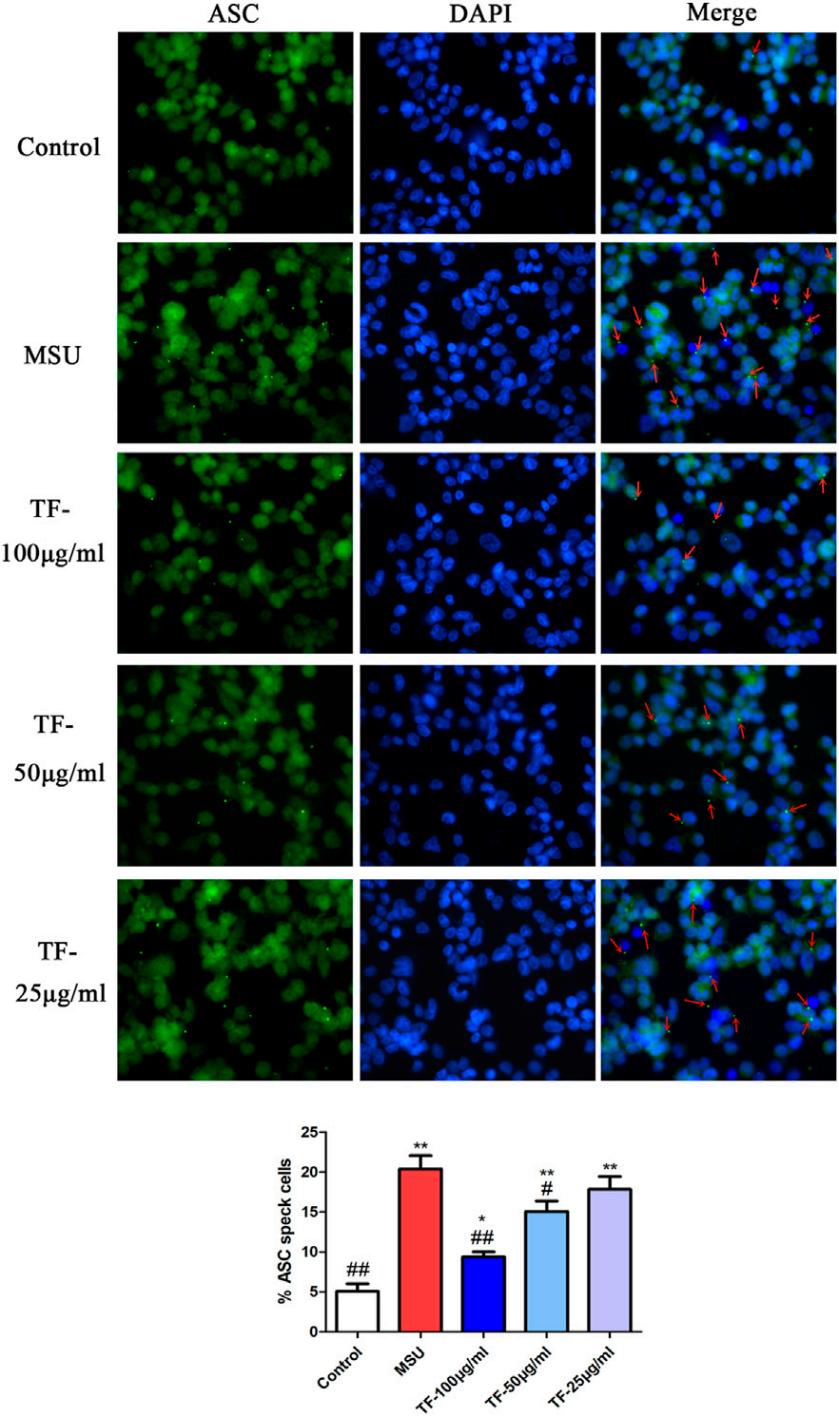
Representative immunofluorescence images of ASC speckle. ASC (green) and DNA (blue). Arrow indicates ASC speckle. *p < 0.05 and **p < 0.01, significantly different the normal control group; ^#^ *p* < 0.05 and ^##^ *p* < 0.01, significantly different the LPS/MSU control group. TF, flavonoid extract.

**FIGURE 9:**
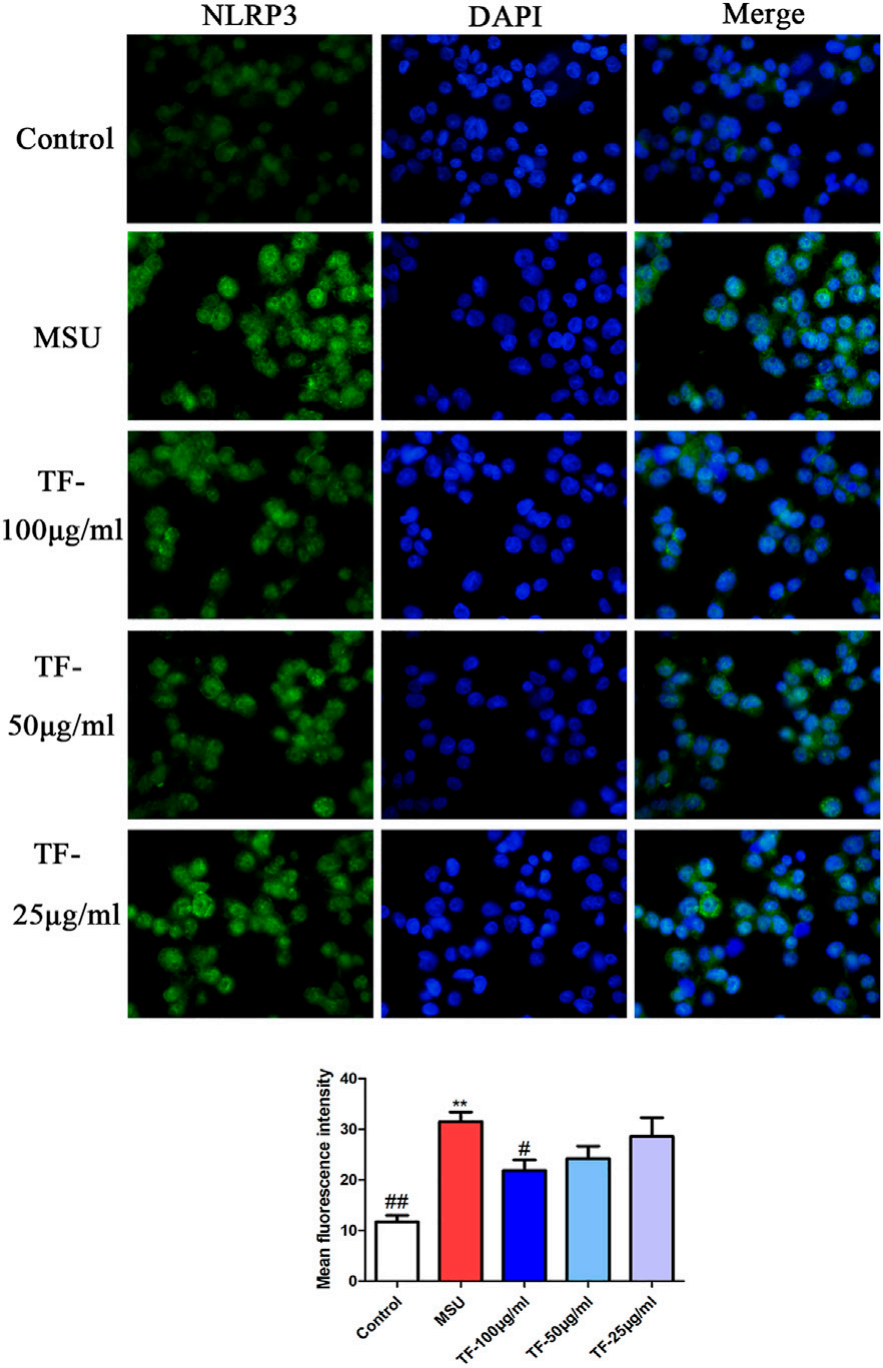
Representative immunofluorescence images of NLRP3 protein. NLRP3 (green) and DNA (blue). *p < 0.05 and **p < 0.01, significantly different the normal control group; #*p* < 0.05 and ##*p* < 0.01, significantly different the LPS/MSU control group. TF, flavonoid extract.

ASC oligomerization is essential for NLRP3 inflammasome activation (Fernandes-Alnemri et al., 2007). As detected in THP-1 macrophages by immunofluorescent staining, ASC oligomerization to form large protein speckles at the perinuclear region was activated by LPS/MSU (Figure 8). Treatment of LPS/MSU-induced THP-1 macrophages with TF markedly reduced the formation of ASC speckles. Besides, the fluorescence intensity of NLRP3 protein was significantly enhanced by MSU (Figure 9). Indeed, pretreatment with 100 μg/mL TF followed by MSU treatment significantly reduced the fluorescence intensity (*p* < 0.05). The immunofluorescence result was consistent with NLRP3 mRNA in terms of lowering NLRP3 expression.

### Effects of TF on IL-1β and Caspase-1 Expression in LPS/MSU-Induced THP-1 Macrophages

**FIGURE 10:**
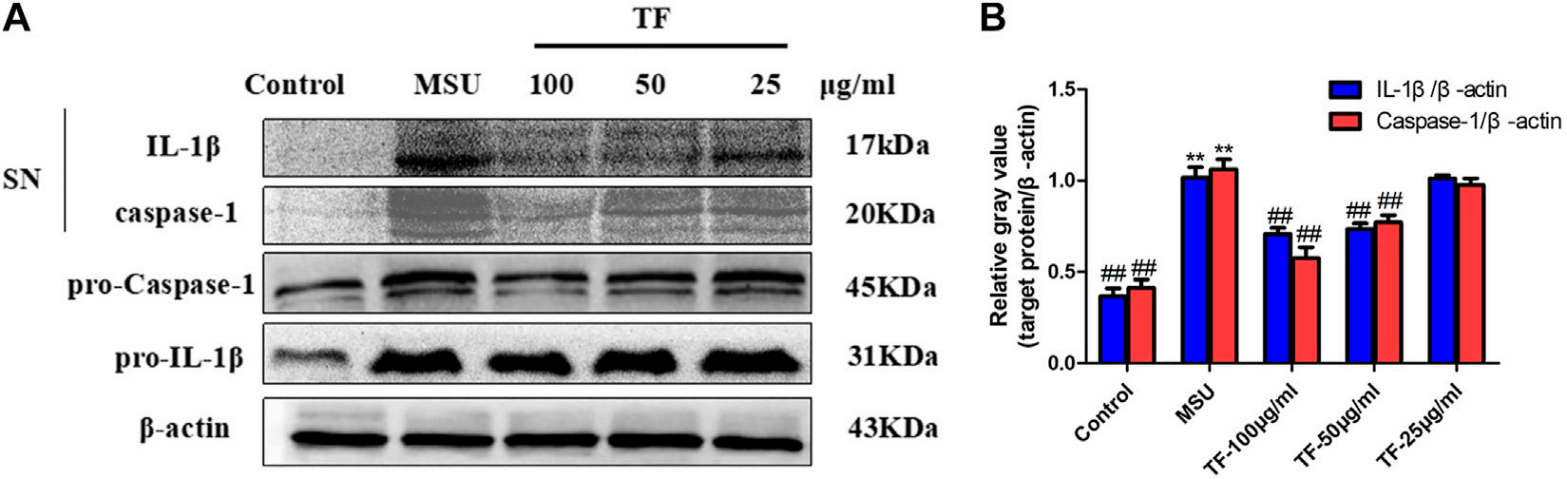
Effects of TF on IL-1β and caspase-1 expression in LPS/MSU-induced THP-1 macrophages. **(A)** Immunoblot analysis of IL-1β and active caspase-1 in supernatants and cell lysates of THP-1 macrophages treated with TF. **(B)** Relative gray value of target protein over β-actin. *p < 0.05 and **p < 0.01, significantly different the normal control group; ^#^ *p* < 0.05 and ^##^ *p* < 0.01, significantly different the LPS/MSU control group. SN, medium supernatants; TF, flavonoid extract.

Cleavage of the precursors proteins pro-caspase-1 and pro-IL-1β to caspase-1 (p20) and IL-1β (p17), respectively, is considered another hallmark of NLRP3 inflammasome activation. By western blot, the amount of active caspase-1 p20 subunit was dose-dependently reduced in supernatants from 50 μg/ml and 100 μg/mL TF-treated macrophages (Figure 10), suggesting that TF inhibits the activation of caspase-1 by NLRP3. Correspondingly, the biologically active p17 form of IL-1β was inhibited by TF, consistent with the ELISA assay's data. Moreover, TF treatment consistently decreased the expression of pro-IL-1β or pro-caspase-1 in cell lysates as well.

### Effects of TF and AM on Gout in MSU-Induced Mice

**FIGURE 11:**
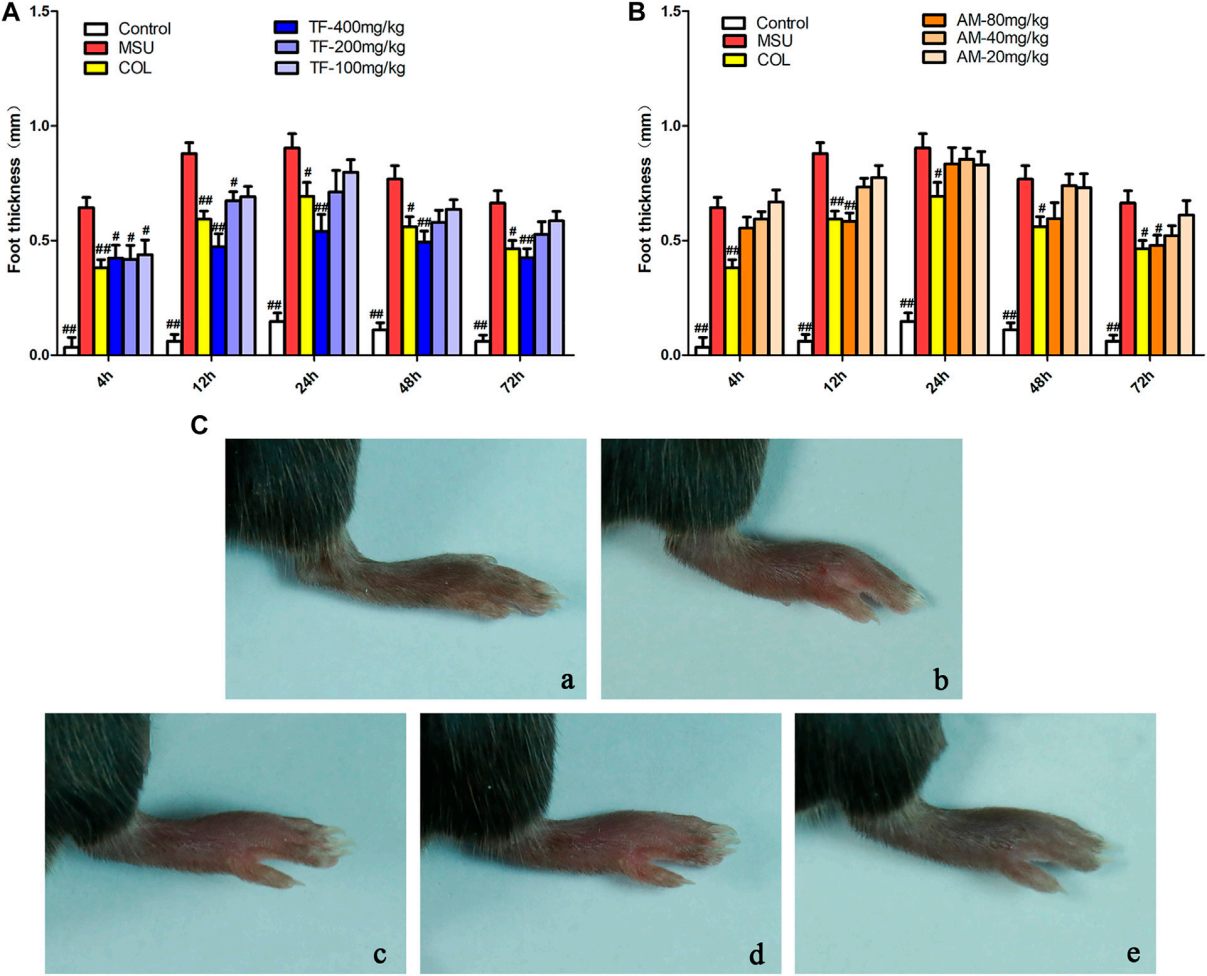
Effects of TF and AM on paw swelling in MSU-induced mice. **(A, B)** Time course of footpad thickness gain compared with the footpad thickness at the 0 h time point per group; **(C)** Paw joint inflammation severity was observed macroscopically. a, Normal control group; b, MSU control group; c, colchicine group; d, TF-400 mg/kg group; e, AM-80 mg/kg group. *p < 0.05 and **p < 0.01, significantly different the normal control group; ^#^ *p* < 0.05 and ^##^ *p* < 0.01, significantly different the LPS/MSU control group. TF, flavonoid extract; AM, amentoflavone.

**FIGURE 12:**
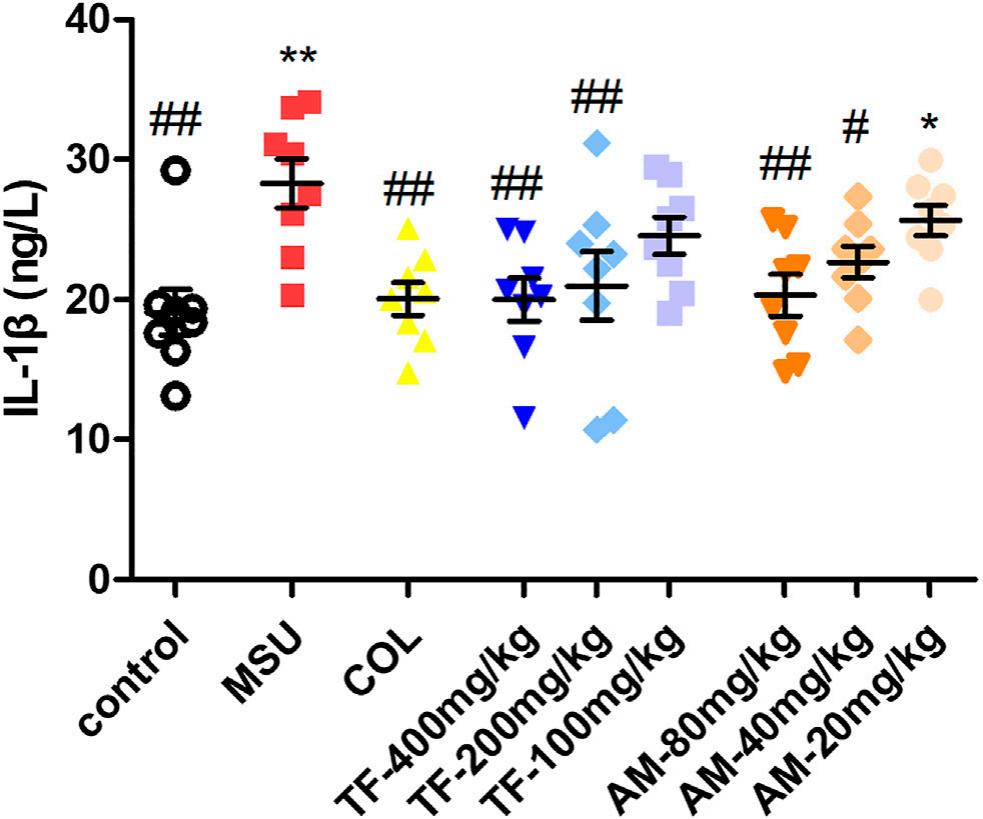
Effects of TF and AM on IL-1β level in MSU-induced mice. Supernatants of the foot tissue homogenates were analyzed for IL-1β after TF treatmentand AM treatment. *p < 0.05 and **p < 0.01, significantly different the normal control group; ^#^ *p* < 0.05 and ^##^ *p* < 0.01, significantly different the LPS/MSU control group. TF, flavonoid extract; AM, amentoflavone.

**FIGURE 13:**
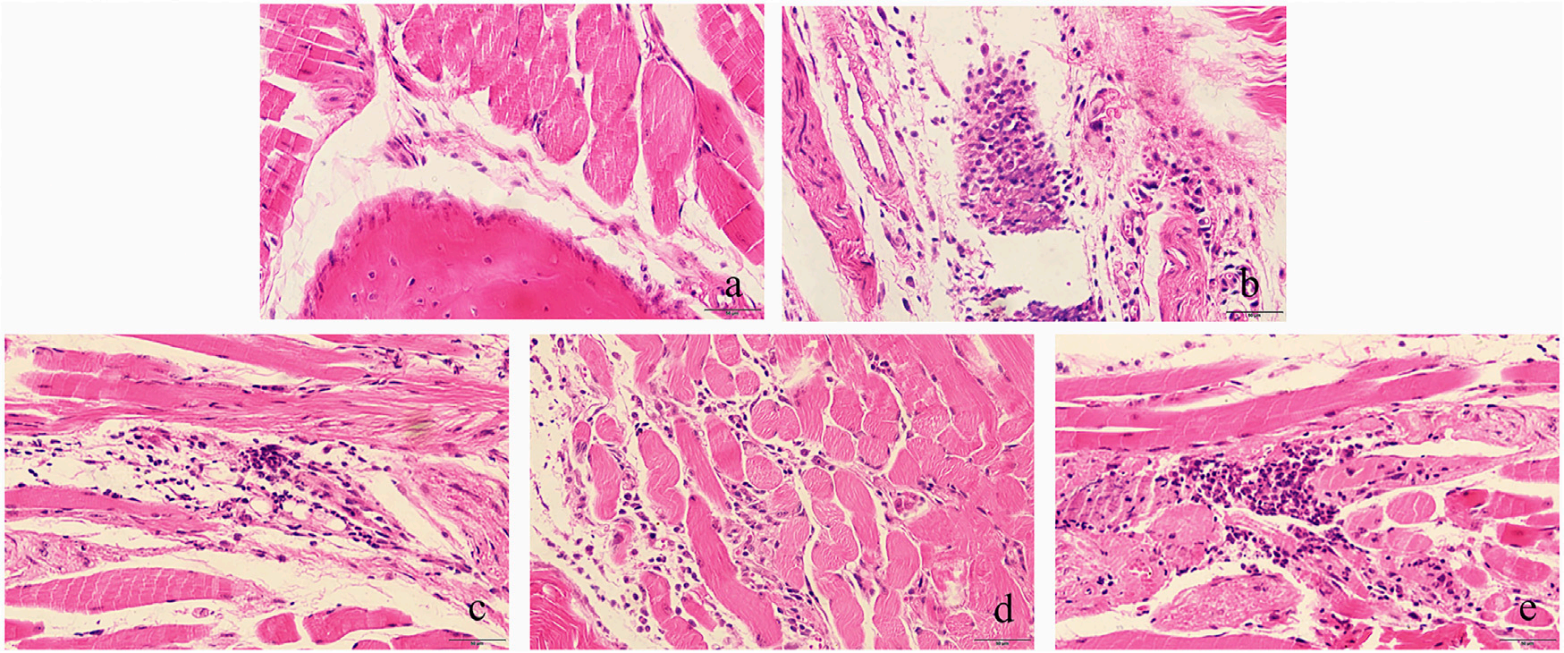
Representative picture of HE staining of the right footpad at plantar side (200×). Infiltrated neutrophils in the hind foot tissue appear as purple dots in HE staining. **(A)** , Normal control group; **(B)** , MSU control group; **(C)** , colchicine group; **(D)**, TF-400 mg/kg group; **(E)**, AM-80 mg/kg group. TF flavonoid extract; AM, amentoflavone.

We measured the paws' thickness after MSU crystal injections. Consistent with the observed increase in paw swelling (Figure 11C), the MSU-induced mice led to a significant increase in foot thickness compared to the control group. This effect reached the peak at 24 h, and the foot thickness increased 0.90 ± 0.19 mm after injection (Figure 11A,B). However, TF and AM administration ameliorated MSU-induced paw edema in a dose-dependent manner to a certain extent (*p* < 0.05, *p* < 0.01). Especially, TF (400 mg/kg) exhibited significantly inhibited MSU-induced paw edema at all evaluated time points, similar to that of colchicine (2 mg/kg, *p* > 0.05). Meanwhile, the IL-1β level of the TF and AM group was also effectively reduced compared to the MSU group (*p* < 0.05), and the effect of 400 mg/kg TF and 80 mg/kg AM were comparable to the colchicine treated group (Figure 12). Histological assessment of the control group revealed the footpad's typical architecture with regular cell arrangements and the absence of abnormal inflammatory cells in the skeletal muscle tissue, fibrotendinous tissue, and subcutaneous tissue (Figure 13A). In the model group, inflammatory cell infiltrations, erosion, and congestion in the subcutaneous soft tissue could be seen from the footpad tissue section (Figure 13B). In contrast, TF and AM with the high dosage treatment could visibly suppress the influx of inflammatory cells, similarly to the colchicine group (Figures 13C–E).

## Discussion

Recently, the development of anti-gout drugs from natural products has attracted worldwide attention for its greater safety and effectiveness. Flavonoids, a class of low or no toxic compounds contrasting to non-steroidal anti-inflammatory drugs, are ideal candidates for gouty arthritis with anti-inflammatory and XOD inhibitory activity. Chemical constituents previously reported of S. moellendorffii included flavonoids, alkaloids, and phenylpropanoids. In our previous research, AM, 4”-O-methylrobustaflavone, 6,8-di-C-β-d-glucopyranosyl apigenin, 6-C-β-d-glucopyranosyl-8-C-β-d-xylopyranosyl apigenin, and 6-C-β-d-xylopyranosyl-8-C-β-d-glucopyranosyl apigenin were isolated from this plant (Zhu et al., 2008). We then investigated these flavonoids' anti-inflammation activities by examining NO secretion of RAW264.7 cells in response to LPS. We found that flavonoid aglycones played a more significant anti-inflammatory activity *in vitro* (data not shown here). In this study, TF purified from ethyl acetate extract was rich in flavonoid aglycones, and AM was its main component. Further research revealed that TF and AM both exhibited remarkable anti-inflammation activity by suppressing NO, IL-1β, and TNF-α production in the cell inflammation model. Previous reports have shown that AM had anti-inflammation, anti-oxidation, anti-tumor, and anti-virus functions (Yu et al., 2017), and inhibiting IL-1β and ICAM-1 by AM may lead to gout suppression in MSU-induced HUVECs (Chen et al., 2016a). Our results further demonstrate that AM is effective in preventing acute gout. Additionally, Jo et al. (2019) reported that robustaflavone could strongly reduce the production of NO, IL-1β, and IL-6 at the concentration of 10 μM. Therefore, we deduced AM and robustaflavone might be responsible for the anti-inflammatory effect of TF.

Activation of NLRP3 inflammasome is the core event of gout. NLRP3 inflammasome activation involves two steps. The first signal, also known as the priming signal, activates the NF-κB pathway, inducing the expression of pro-IL-1β and NLRP3 mRNA. The second step, activation signal, is transduced by various PAMPs and DAMPs, resulting in the formation of the inflammasome complex and the subsequent activation of caspase-1 (Place and Kanneganti, 2018). Here, pretreatment with TF and AM significantly inhibited the IL-1β secretion and caspase-1 cleavage in a dose-dependent manner. These results suggested that TF inhibited NLRP3 inflammasome activation. ASC on mitochondria bridges NLRP3 and caspase-1 to form NLRP3 ternary inflammasome complexes (Compan et al., 2015). However, treatment with TF markedly reduced ASC speckles' formation, suggesting TF may block the assembly of NLRP3 inflammasome characterized by less formation of ASC speckles and expression of NLRP3. Activation of caspase-1 not only leads to inflammation but in certain instances causes an inflammatory form of cell death called pyroptosis (Fink and Cookson, 2006). It is characterized by cell swelling, lysis, and release of pro-inflammatory cytokines and intracellular contents such as LDH (Fink and Cookson, 2006). However, we further found that TF could significantly reduction LDH release and the percentage of PI-positive cells. By SEM, THP-1 cells that were exposed to 100 μg/mL TF exhibited pronounced cell rounding with a few small extended lamellipodia. The data suggested that TF pretreatment inhibited MSU-induced cell inflammation by improving cell membrane integrity and protecting cell morphology. Therefore, the results indicated that TF alleviated LPS/MSU induced cellular gout model via regulation of NLRP3/ASC/Caspase-1 signaling pathway.

Intradermal injection of MSU crystals into the footpad has been regarded as a classical model to reproduce gouty arthritis experimentally. After taking in the MSU crystals, the secretion of IL-1β and TNF-α via monocytes is promoted. IL-1β has emerged as an important indicator to assess the acute gout model (So et al., 2018). The accumulation of IL-1β causes synovial lesions, cartilage destruction, and neutrophil infiltration. Interestingly, TF and AM could markedly relieve paw swelling and decrease IL-1β levels. As further positive evidence, administration of TF and AM successfully alleviated the pathologic changes of the footpad in mice injected with MSU as detected by HE staining. These successfully confirmed that TF and AM had good effects on acute gouty arthritis by reducing foot thickness, IL-1β level, and lymphocyte infiltration.

Collectively, TF may influence the NLRP3 inflammasome activation by inhibiting mRNA levels of IL-1β, TNF-α, NLRP3, and caspase-1, reducing the formation of ASC speckles and decreasing the expression of NLRP3, whereas inhibiting downstream signal molecular IL-1β release and caspase-1-dependent cell pyroptosis. Our results further confirmed that TF and its main constituent AM could prevent MSU-induced gout *in vivo*. Thus, TF may have preventive or therapeutic potential against acute gout via regulation of NLRP3/ASC/Caspase-1 signaling pathway.

## Data Availability

The original contributions presented in the study are included in the article/Supplementary Material, further inquiries can be directed to the corresponding author.
